# Light Adaptations of *Ipomoea purpurea* (L.) Roth: Functional Analysis of Leaf and Petal Interfaces

**DOI:** 10.3390/plants14060862

**Published:** 2025-03-10

**Authors:** Zhanlin Bei, Lulu Lu, Zubayda Amar, Xin Zhang

**Affiliations:** 1 School of Biological Science and Engineering, North Minzu University, Yinchuan 750021, China; realpal00147@163.com (Z.B.); luludyx0916@163.com (L.L.); zubaidan0417@163.com (Z.A.); 2 Key Laboratory of Ecological Protection of Agro-Pastoral Ecotones in the Yellow River Basin, National Ethnic Affairs Commission of the People’s Republic of China, Yinchuan 750021, China

**Keywords:** *Ipomoea purpurea*, light adaptations, leaf and petal interfaces, pollinator attraction, self-cleaning

## Abstract

In low-light environments, plants face challenges in maximizing light acquisition for growth and reproduction. This study investigates the light-related adaptations of *Ipomoea purpurea* (L.) Roth, a climbing annual vine commonly known as morning glory. Field and laboratory analyses focused on the functionality of its leaf and petal interfaces. We observed that tendrils of *I. purpurea* enable it to climb surrounding structures, optimizing light capture. The leaves display absorption peaks at 400 nm and 700 nm, typical for plants that absorb light in the red and blue regions, with microstructural features like protrusions and folds aiding in self-cleaning. Petals, exhibiting grid-like patterns and specific reflectance spectra, attract pollinators such as bees. These functional traits, including self-cleaning mechanisms and specialized light absorption, highlight *I. purpurea*’s unique strategies for thriving in low-light conditions. The findings offer valuable insights into the potential use of *I. purpurea* for urban landscaping, vertical greening, and ornamental plant selection.

## 1. Introduction

Green plants efficiently utilize solar energy in sufficient light conditions for photosynthesis and transpiration, such as synthesizing essential organic compounds and regulating temperature through heat dissipation. Various physiological and morphological adaptations have evolved to optimize light acquisition [1]. Under ideal conditions, plants convert only about 10% of absorbed light energy into glucose [2]. However, in low-light environments, their capacity to regulate light absorption is limited [3]. Despite this, plants exhibit adaptive behaviors such as heliotropism, where leaves orient to capture sunlight [4].

Plant heliotropic behavior usually occurs under conditions that favor increased photosynthesis, growth, survival, and competitive advantage [5]. In different habitats, the organs of plants exhibit varying degrees of plasticity [2]. Plants living in areas with abundant sunlight but limited moisture utilize their leaf surface trichomes to filter sunlight and capture concealed moisture from the air [6,7,8]. In alpine snowy environments, the petals of plants track sunlight to increase pollen deposition, thereby enhancing their attractiveness to pollinators [9]. Under conditions where moisture and temperature permit, the orientation of plant organs continuously adjusts throughout the day to increase the temperature of the plant reproductive organs and basking insects [10]. To “track” sunlight, plants not only rotate to face the sun but may also climb on objects such as walls and trees to reach higher positions [11], ensuring the acquisition of more light energy [12].

Climbing plants employ diverse strategies to optimize light acquisition in different environments. *Ipomoea purpurea*, like many heliotropic species, exhibits solar tracking to maximize light interception throughout the day [13]. In contrast, *Phaseolus vulgaris*, a climbing legume, primarily relies on leaf orientation adjustments, such as petiole movement, to optimize light capture [14]. Shade-adapted plants, including certain understory ferns and herbs, follow alternative strategies: they often possess a high leaf area index (LAI) and an increased proportion of chlorophyll b to enhance low-light utilization efficiency [15]. Additionally, some shade plants have evolved specialized epidermal cells functioning as lenses to focus incoming light and improve photosynthetic efficiency [16]. Structural adaptations such as thin leaves with higher chlorophyll content and red abaxial cell layers further enhance light capture in low-light environments [17]. These comparisons provide a broader ecological context for understanding *I. purpurea*’s unique light adaptation strategies and their implications for biomimetic applications.

However, for plants growing in low-light environments under the forest canopy, leaves and petals face practical challenges in utilizing light effectively: they need vibrant petals to attract major pollinators such as bees, while leaves serve as solar panels for light harvesting and heat dissipation plates for temperature regulation. Maintaining a dust-free leaf surface is crucial for normal physiological activities, especially for plants with a limited number of leaves. In low-light environments, such as under the forest canopy, plants face challenges in optimizing light use. Leaves must function efficiently as solar panels for light harvesting and heat dissipation, while petals must be vibrant enough to attract pollinators like bees. Maintaining a dust-free leaf surface is crucial for normal physiological activities, especially for plants with limited leaf numbers. Studies by Van Der Kooi et al., (2021) suggest that insect pollinators, particularly bees, are drawn to the UV reflectance in floral petals, influencing plant-pollinator interactions [18]. Similarly, research by Suárez-Cáceres et al., (2023) and Zhou et al., (2024) highlights how plant performance in urban greening systems, such as green roofs and living walls, is strongly influenced by growth and surface characteristics [19,20]. Over evolutionary time, plants have continuously optimized their structures and functions to adapt to specific habitats [20]. Bio-inspired design (BID) extracts functional principles from biological systems and applies them to technological fields, where understanding biological adaptations is crucial for efficient BID [20]. The structural features of plant surfaces play a significant role in optimizing light absorption and maintaining cleanliness, inspiring research on biomimetic materials and self-cleaning surfaces.

This study focuses on the annual vine plant, *Ipomoea purpurea* (L.) Roth, commonly known as morning glory. The native range of *I. purpurea* is tropical and subtropical America, and it possesses significant ecological and medicinal value. Despite having a limited number of leaves, morning glory plants utilize tendrils to spiral upward around plant stems and tree trunks, allowing leaves and petals to capture more light energy. This raises an important question: how does morning glory maximize light acquisition with a limited number of leaves and petals? Long-term field observations revealed that the leaves and flower surfaces of *I. purpurea* remain clean, prompting us to investigate the underlying structural characteristics. This study explores the mechanisms behind this phenomenon by focusing on the external structures of the leaves and flowers, which may provide bio-inspired insights for optical materials or energy harvesting.

## 2. Results

### 2.1. Morphological Characteristics of Leaves and Petals

**Figure 1:**
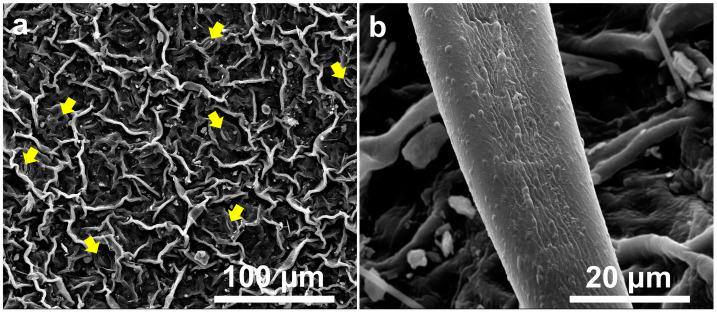
Scanning Electron Micrograph of *I. purpurea* Leaf. (**a**). Stomata present on leaf surface (indicated by yellow arrows); (**b**). Sparse trichomes present on leaf upper surface.

**Figure 2:**
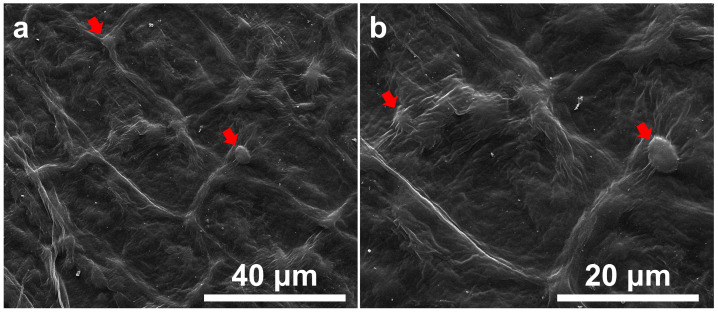
Scanning Electron Micrograph of *I. purpurea* Petal. (**a**). Surface exhibits grid-like raised ridges; (**b**). Protrusions are present at intersections (indicated by red arrows).

The leaves of *I. purpurea* exhibit 5–20 µm protrusions and folds, with stomata present on the surface (Figure 1a). Sparse trichomes also cover the leaf surface (Figure 1b). The surface of the petals displays raised, grid-like structures with protrusions at intersections (Figure 2).

### 2.2. Contact Angle of Leaves and Petals Interfaces

**Figure 3:**
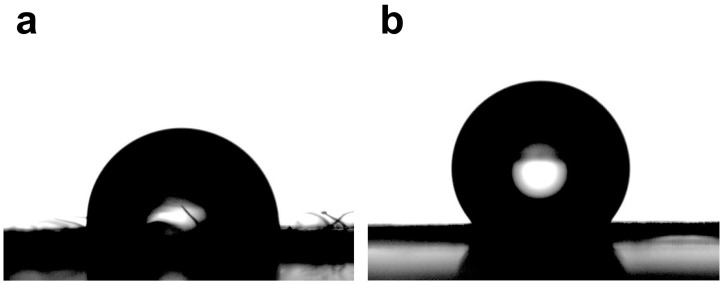
The Wetting of Leaf and Petal Interfaces in *I. purpurea*. (**a**). Contact angle of *I. purpurea* leaf (92.13 ± 3.05°); (**b**). Contact angle of *I. purpurea* petal (127.02 ± 6.30°).

Contact angle measurements assessed the wetting behavior of water droplets on *I. purpurea* leaves and petals. Results indicate that water droplets form a spherical cap on the leaf surface, with a contact angle of 92.13 ± 3.05° (*n* = 5), suggesting slight hydrophobicity. This hydrophobicity aids self-cleaning, which is crucial for maintaining efficient photosynthesis in low-light environments. On the petal surface, water droplets form a spherical shape with a contact angle of 127.02 ± 6.30° (*n* = 5), indicating a greater degree of hydrophobicity compared to the leaf (Figure 3). These findings highlight the adaptive features of *I. purpurea* in its natural habitat.

### 2.3. Reflection Spectra of Leaf and Petal Interfaces

**Figure 4:**
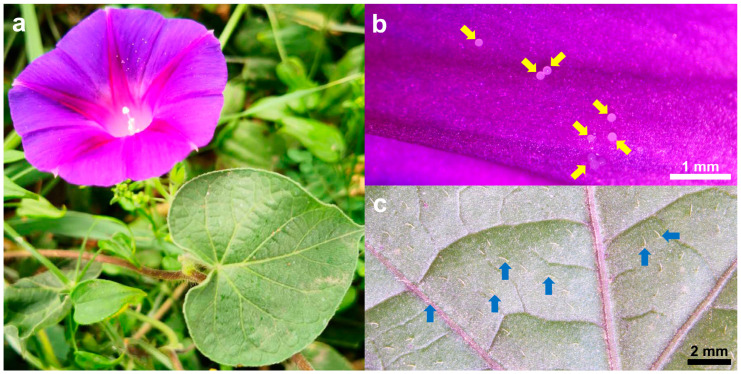
Optical Images of *I. purpurea* Leaf and Petal. (**a**) Optical image of the leaf and petal of *I. purpurea*. (**b**) Optical microscopy image of the petal surface. The yellow arrows in the figure indicate the pollen grains. (**c**) Optical microscopy image of the leaf surface. The blue arrows in the figure indicate the trichomes.

**Figure 5:**
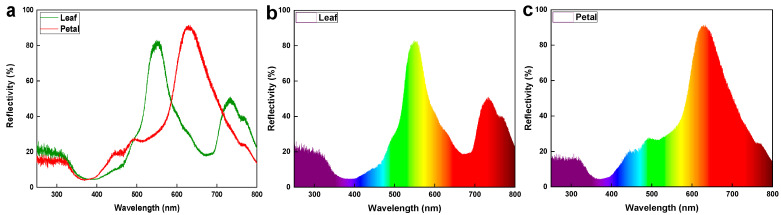
(**a**) Reflectance spectra of *I. purpurea* leaf and petal. The green line in the legend represents the reflectance spectrum of the leaf.The red line in the legend represents the reflectance spectrum of the petal. (**b**) Specific light colors in the reflectance spectra of *I. purpurea* leaves. (**c**) Specific light colors in the reflectance spectra of *I. purpurea* petals. The colors in figures b and c correspond to the color of light at the wavelengths along the x-axis. Wavelengths below 380 nm represent ultraviolet light. The y-axis represents the relative reflectance of the sample at each specific wavelength.

**Figure 6:**
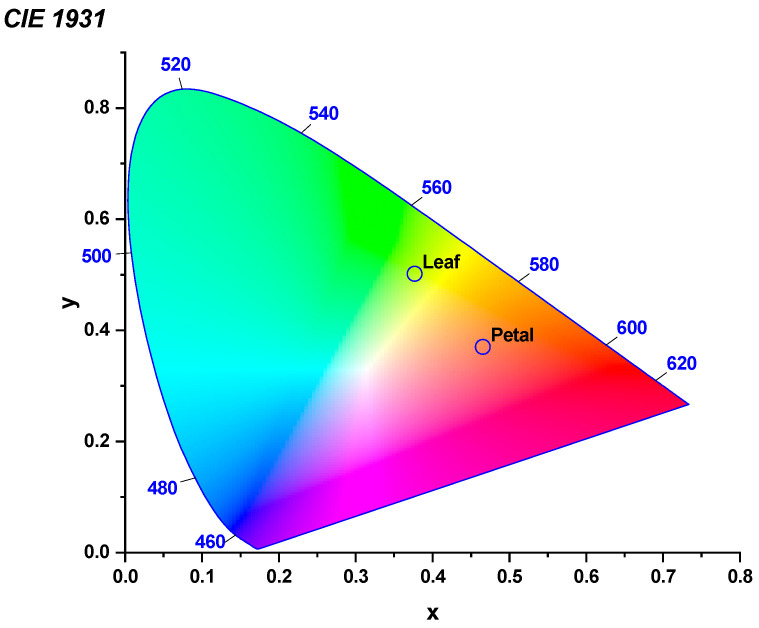
Chromaticity Diagram of *I. purpurea* Leaves and Petals. In the CIE 1931 color space, the coordinates of **x** and **y** range from (0, 0) to (1, 1), describing a wide range of colors from purple to red, green, and blue. The x coordinate represents the horizontal position of the color in the diagram, and it is related to the red component of the color. Higher **x** values generally correspond to more reddish colors. The **y** coordinate represents the green component of the color, and together with x, it determines the position of the color in the chromaticity diagram. The circles in the diagram represent the colors perceived by the human eye for the leaf and petal.

**Figure 7:**
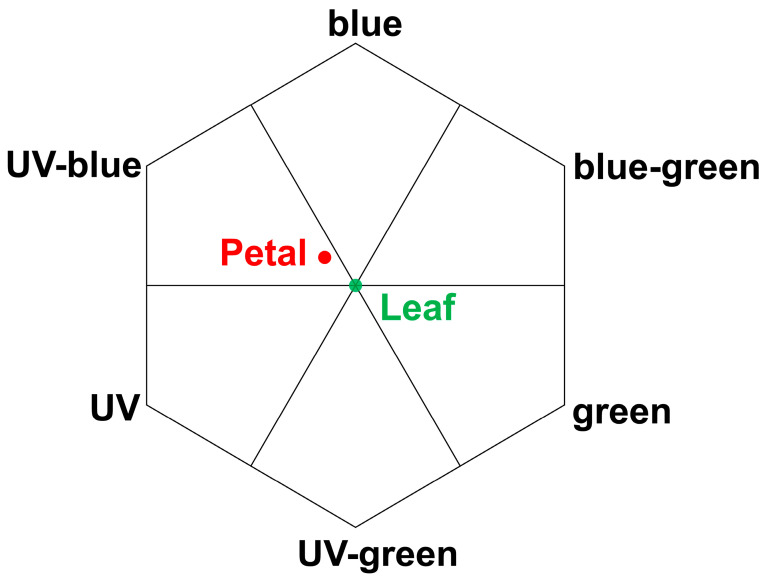
Hexagon Model of Bee Visual Perception for Color Positions of *I. purpurea* Petals.

The surface reflectance spectra of *I. purpurea* leaves and petals were measured in the UV-visible-near infrared regions. Different absorption and reflection peaks were observed for leaves and petals across different wavelengths (Figure 4 and Figure 5). Leaves exhibited absorption peaks near 400 nm and 700 nm, with a strong reflection peak near 550 nm and 730 nm (Figure 5), while petals showed absorption peaks near 400 nm and a strong reflection peak near 630 nm (Figure 5), with the wavelengths of reflection clarified to correspond to green and purple. Colorimetric diagrams (CIE) and hexagonal models of bee vision color space corresponding to petal colors were constructed based on the spectral reflectance curves (Figure 6 and Figure 7).

## 3. Discussion

The study found that the leaves of *I. purpurea* demonstrated slight hydrophobicity (Figure 3a), while the petals more pronounced hydrophobicity (Figure 3b). The leaves of *I. purpurea* could absorb and utilize the physiologically active portions of the solar continuous spectrum, particularly in the red and blue light bands, with high reflectance in the ultraviolet and infrared regions (Figure 5a,b). The petals of *I. purpurea* absorbed light in the blue light band and exhibited high reflectance in the red light region, displaying the color of the petals within the visible light range (Figure 5c). The bee vision color hexagon model demonstrated that the color points of *I. purpurea* petals stimulated the blue and ultraviolet light regions in the compound eyes of bees (Figure 7).

**Figure 8:**
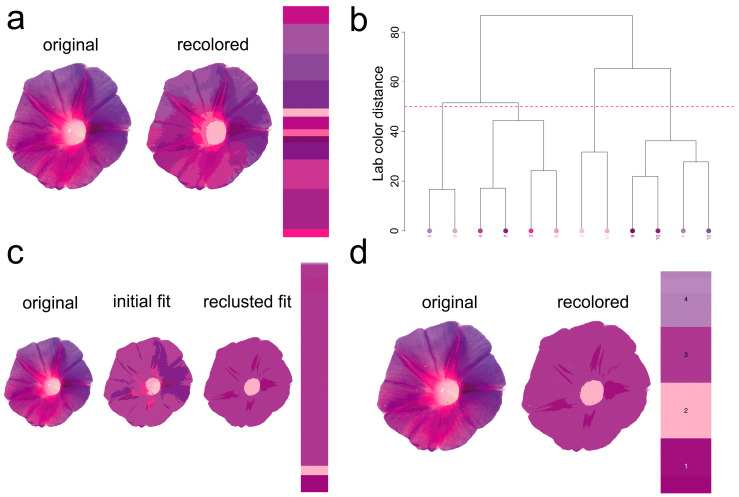
Image of *I. purpurea* Petal, Demonstrating the Core Steps of the Package. (**a**). First, the original image’s pixels are grouped within each axis of color space, using a user-defined number of bins per channel through the recolorize function. (**b**). These bins are then merged based on a rule—specifically, the distance in CIELAB space, calculated using the recluster function. Bins with a Euclidean distance smaller than the user-selected threshold (cut-off = 50) are combined, and the image is refitted using the resulting set of color centers. (**c**). The zone map is generated, showing the original, initial fit, and reclustered fit. (**d**). Individual color patches are then exported as binary masks using the splitByColor function. The final segmented color patches represent the colors as perceived by humans.The colored dots in Figure b represent different color samples segmented from the petal using k-means clustering, with each number corresponding to a distinct color cluster. These samples are analyzed in the CIELAB color space, where the distance between the color samples is calculated. The closer the colors are in the CIELAB space, the more similar the samples are in color perception.In Figure d, the re-colored colors of the petal are divided into 4 categories (i.e., 4 color clusters), with each number representing one color cluster.

Currently, *I. purpurea* is a widely cultivated ornamental flower globally, commonly used for vertical greening in fences, balcony windowsills, railings, and pergolas. Also known as morning glory, it belongs to the Convolvulaceae family and is an annual climbing herbaceous plant, loved by people of all ages [21]. The corolla of *I. purpurea* is as thin as gauze, and its petals unfurl gracefully at dawn. Because it opens towards the sun, it is also called “Asagao” (morning glory) in Japan [22]. As a climbing plant, *I. purpurea* can also be planted along sidewalks, with lush foliage and a flowering period lasting 4 to 5 months, playing a role in greening and beautification. The leaves are sparsely or densely covered with soft hairs, contributing to oxygenation, cooling, dust retention, and noise reduction [23]. In addition, in some cultures, colors carry specific meanings, such as purple representing love, holiness, and honor [24]. This study examined the spectral reflection characteristics of *I. purpurea* leaves and petals and conducted wettability tests on them. It analyzed *I. purpurea*’s ability to climb and compete for light using its tendrils, the attraction of its petal color to insects, the characteristics and functions of leaf and petal interfaces (Figure 7), and the human-subjective perception of petal color pattern geometry. The petals of *I. purpurea* are primarily purple (Figure 8).

The dispersion of dust in the atmosphere is a common phenomenon in industrialized cities, especially in areas with no dominant wind direction, where dust is not easily dispersed, leading to poor air quality. If the relative humidity near the ground is high and there is much on the ground, the particles in the atmosphere tend to stabilize, resulting in haze formation [25]. A haze is a hazardous meteorological phenomenon where tiny particles float in the air [26]. Atmospheric dust not only affects the healthy growth of vegetation but also harms ecosystems. Moreover, atmospheric dust, which carries a wide variety of heavy metal particles, is one of the most harmful components in the air and can severely affect human health [27]. Plants, with their special canopy structure and the trichomes on their leaf surfaces, can attract, adhere to, and thereby filter a certain amount of suspended dust particles in the air, thereby reducing atmospheric dust particles [28]. Particularly, plants used in landscaping and vertical greening can effectively block the spread of suspended dust, thus positively improving atmospheric dust deposition [29].

Wettability is one of the important characteristics of solid surfaces, determined by the surface’s chemical composition and micro-geometric structure [30]. The static contact angle (CA) of water droplets on a solid surface is commonly used to measure surface wettability. When CA is less than 90°, or less than 5°, it is respectively referred to as hydrophilic or superhydrophilic materials; when CA is greater than 90°, or greater than 150°, it is respectively referred to as hydrophobic or superhydrophobic materials [31]. As a plant used in vertical greening, the *I. purpurea* has trichomes on its leaves, which can effectively trap particles in the air. In this study, the wettability of *I. purpurea* leaves was determined by measuring the contact angle on the leaf surface, resulting in a contact angle of 92.13 ± 3.05°, indicating slight hydrophobicity. The leaf surface also exhibits a certain degree of self-cleaning property, as dust adhered to the surface can be removed by rainwater droplets rolling off the leaf surface. The contact angle on the petal surface was measured to be 127.02 ± 6.30°, indicating hydrophobicity.

In the study, it was found that during photosynthesis, green leaves primarily absorb and utilize the physiologically active portions of the solar spectrum (blue light 400 nm to 520 nm and red light 610 nm to 720 nm), while exhibiting strong reflection and minimal absorption of the near-infrared light portion (700 nm to 1400 nm) [32]. Infrared light absorbed by water molecules within the plant generates heat, driving transpiration by releasing free water molecules [33,34]. Red light also affects plants’ sun-tracking behavior [35]. *I. purpurea* leaves exhibit absorption in red and blue light ranges, while showing high reflection in the ultraviolet and infrared regions. Petals of *I. purpurea* absorb blue light and reflect strongly in the red light range, displaying the color of the petals within the visible light spectrum. Studies have shown that low-intensity laser radiation can stimulate the synthesis of these compounds, enhancing the plant’s antioxidant properties and resistance to abiotic stressors [36,37,38]. This suggests that similar mechanisms may underlie the light absorption abilities observed in *I. purpurea* leaves.

**Figure 9:**
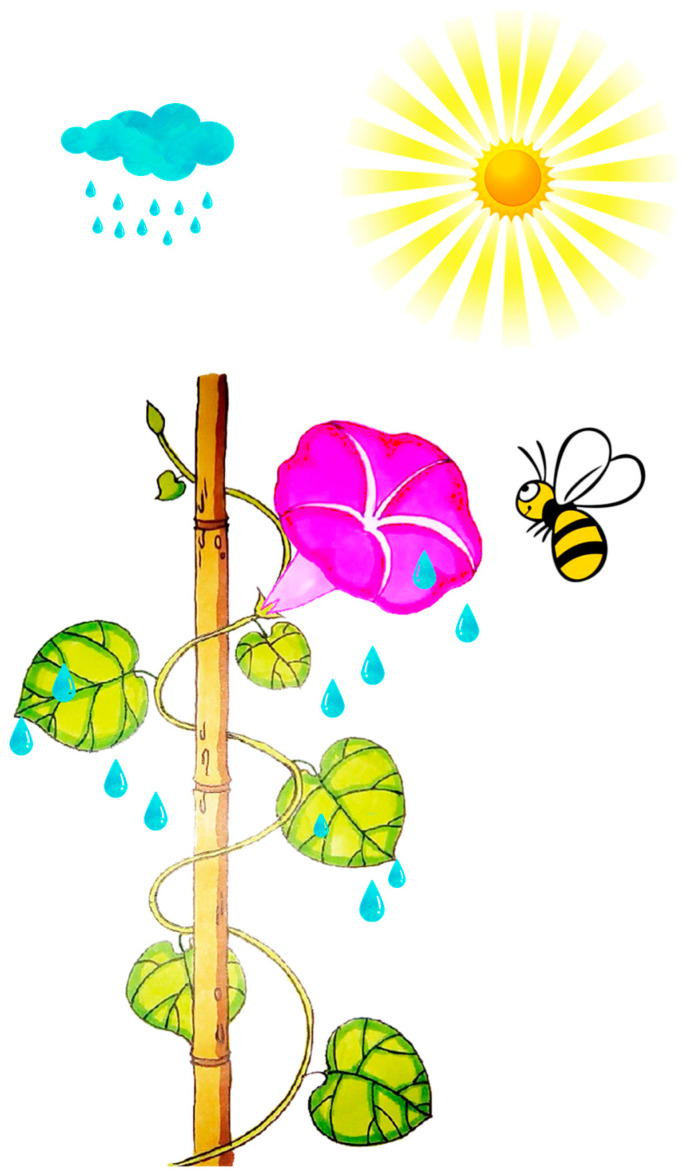
Response Patterns of *I. purpurea* Leaves and Petals to Light, Water, and Bees.

Plants typically utilize various cues such as odor [39], color [40], and shape [41] to attract and assist important pollinators in locating flowers. Among these, pollinators rely on the color characteristics based on the flower’s reflectance spectrum. For instance, hymenopterans (bees) possess three types of photoreceptors in their compound eyes: ultraviolet (UV) photoreceptors, blue (Blue) photoreceptors, and green (Green) photoreceptors, with the highest sensitivity to light at wavelengths of 340 nm [42], 430 nm [43], and 540 nm [44], respectively [45]. The morning glory, as an annual, self-compatible vine, exhibits high resistance to herbivorous insects and relatively low damage tolerance [46]. *I. purpurea* corolla can open and close. The opening and closing of the *I. purpurea* corolla is an important physiological trait for environmental adaptation, involving both cellular morphological changes and complex transcriptional regulation [47]. Studies have shown that corolla closure is primarily controlled by the collapse of bulliform cells in the adaxial epidermis and the bending of acuminate veins. These morphological features may influence light capture and habitat adaptation, providing insights for further research on their optical functions [47].Additionally, morning glory flowers display significant polymorphism in color, typically exhibiting eight color phenotypes. The variation in flower color has been shown to influence the behavior of non-herbivorous pollinators and the outcrossing rate of morning glory [48]. The petals of *I. purpurea* tested in this experiment, within the bee’s visual color space hexagon model, showed stimulating reflectance rates in the blue and ultraviolet spectra for bees. The petal surface has edges with protrusions, which facilitate insect landing on the petal surface. Therefore, the petal color of *I. purpurea* is attractive to bees and humans (Figure 2, Figure 4, Figure 7, Figure 8 and Figure 9).

Additionally, studies have reported the carbon sequestration potential of Beach Morning Glory (*Ipomoea pes-caprae*) on green roofs [49]. Reports indicate that green roof plants have significant potential for carbon sequestration [50]. We believe that in this study, *I. purpurea* may have potential carbon sequestration capabilities in improving air quality and reducing CO_2_ concentrations, especially in vertical greening environments.

## 4. Materials and Methods

### 4.1. Leaf and Petal Collection

In July 2019, we collected adult leaves and fully opened petals of *I. purpurea* from Sanshayuan Park, Yinchuan City. The identification of the plants was performed based on morphological characteristics, including leaf shape, flower color, and growth habit, following the taxonomic keys provided in relevant literature [51]. Fresh samples were gently rinsed with distilled water to remove surface impurities and were subsequently used for reflectance spectroscopy and wetting tests.

### 4.2. Observation of Petal and Leaf Morphology

Fresh leaves and petals were washed twice with ultrapure water (UPH-II-10T, Chengdu Ultra Pure Technology Co., Ltd., Chengdu, China) for 5 min each time, followed by dehydration with a series of gradient alcohols (30%, 50%, 70%, 80%, 90%, 95%, and 100%, each for 10 min). The samples were gently attached to conductive adhesive, subjected to gold spraying with an ion sputtering instrument (Hitachi E-1045, High-Tech Corporation, Tokyo, Japan), and observed for leaf surface ultrastructure using a scanning electron microscope (Inspect, FEI, Thermo Fisher, Waltham, MA, USA) with a maximum magnification of 650,000×, an accelerating voltage of 0.530 kV, and a resolution of 2.2 nm at 1 kV and 1.0 nm at 15 kV.

### 4.3. Reflectance Spectroscopy Testing

A USB4000 spectrometer (Ocean Optics, Inc., Dunedin, FL, USA) was used to measure the spectral reflectance of fresh *I. purpurea* leaves and petals in the range of 250 nm to 800 nm. Prior to measurement, the spectrometer was calibrated using a standard white reference panel. Measurements were taken at a fixed viewing angle of 25°. For each sample, three replicates were performed, and the mean value was taken as the spectral reflectance of the sample. Data acquisition and storage were performed using the accompanying OceanView software (Ocean Optics, USB4000).

### 4.4. Wetting Test

The wetting properties of the experimental samples were measured using a video optical contact angle measurement instrument OCA25 (Dataphysics Inc., Filderstadt, Germany). Pure water droplets were used, and contact angle measurements were performed at an environmental temperature of 28 °C and a relative humidity of 65%. The instrument’s built-in high-speed imaging system captured images, and the accompanying software analyzed and processed the contact angle data.

### 4.5. Establishment of Bee Vision Color Hexagon Model

Data from reflectance spectroscopy were analyzed using R 4.4.0 software, constructing a color hexagon model for bee vision [52,53].

### 4.6. Flexible Color Segmentation of Biological Images

The recolorize package (version 0.1.0) [54] provides a range of segmentation tools rather than a single algorithm. Here, we focus on the typical image segmentation process.

First, each pixel in the image of *I. purpurea* (digital photo) is assigned to a color class during an initial binning step using the recolorize function. Instead of k-means clustering (an alternative method in the package), the default approach uses a histogram method, where pixels are grouped based on predefined ranges for each axis of the color space. The total number of color centers is determined by multiplying the number of bins per channel. Each region’s center is calculated as the average of all pixels assigned to it (or the geometric center if no pixels are assigned).

Next, the initial color centers are refined using user-defined methods, such as merging similar centers (via the recluster function) or eliminating small color patches (using thresholdRecolor). The recluster function, which is the most effective for this step, calculates the Euclidean distance between pairs of color centers. These centers are then grouped based on similarity using hierarchical clustering. Users can specify either a similarity threshold or a final number of classes. In this case, we used a cut-off of 50 (Euclidean distance in CIELAB space) to reduce the 12 initial centers to 4 consensus centers. The recluster function then refits the original image with these new centers.

### 4.7. Data Statistics and Analysis

Statistical analysis and plotting of experimental data were performed using R 4.4.0 and OriginPro (Version 2023, OriginLab Corporation, Northampton, MA, USA).

## 5. Conclusions

This study analyzes the reflectance spectroscopy characteristics and wettability of the leaves and petals of the annual vine, *I. purpurea* (morning glory). Our findings indicate that the leaves of *I. purpurea* possess self-cleaning properties that prevent dust accumulation, with significant absorption of red and blue light wavelengths crucial for photosynthesis. Absorption peaks were observed near 400 nm and 700 nm, with a strong reflection peak near 750 nm. Petals exhibited absorption near 400 nm and a strong reflection peak near 600 nm. Reflectance spectra modeling showed that petal colors are highly attractive to bees, indicating their role in pollination. The unique functionalities of the leaves and petals, including micron-scale protrusions and folds, enable *I. purpurea* to thrive in low-light environments. These findings offer important insights for selecting and using ornamental plants in urban landscaping, particularly for enhancing aesthetic appeal and promoting sustainable, three-dimensional greening. Future research should investigate the genetic mechanisms underlying these adaptations, which may have broader applications in improving the performance of other plant species in similar environments.

## Data Availability

The authors declare that the data supporting the findings of this study are available within the paper, or from the corresponding author on reasonable request.
